# Supplementary material for: Are carbon emissions trading and green financial instruments synergistic? -Comprehensive quantitative research based on content analysis
Source: PLoS One. 2024 Mar 7;19(3):e0298601. doi: 10.1371/journal.pone.0298601 (PMC10919601; doi:10.1371/journal.pone.0298601)
Supplement: S1 Data — (ZIP) [file pone.0298601.s001.zip › Data/地级市Z_F工作报告.zip!/╕ú╜¿/╧├├┼╩╨/└┤╫╘╣1⁄2╓┌║┼í╛┬φ┐╦╩2╛▌═°í┐_2005.docx]

各位代表：

　　现在，我代表市人民政府向大会作政府工作报告，请予审议，并请市政协委员和各位列席人员提出意见。

　　一、2004年的工作

　　2004年是全面树立和落实科学发展观，推进海峡西岸经济区建设的第一年。我们在中共中央、国务院，福建省委、省政府和厦门市委的领导下，经过登高人民的共同努力，完成了市十二届人大二次会议确定的预期目标。全年实现生产883.2总值亿元，增长16%；财政总收入160.4亿元（剔除出口退税因素，达180.3亿元），同口径增长20.8%，其中地方级财政收入68.2亿元（剔除出口退税因素，达88.2亿元），同口径增长20.2%；城镇居民人均可支配收入14443元，农民人均纯收入5647元，分别增长11.8%和9.6%；城镇登记失业率4.1%，居民消费价格指数为103.1，人口出生率9.1%。

　　这一年，我们克服了干旱限电、原料燃料价格上涨等困难，积极适应出口退税、土地调控的政策调整，经济发展保持较快速度，质量效益明显搞，发展后劲得到增强，人民收入增幅较大，工业总产值提前一年完成“十五”计划目标，岛外固定资产投资和工业产值与岛内持平，外贸进出口突破200亿美元，空港客运量突破500万人次，海港货物吞吐量突破4000万吨，厦门港被国家定位为国际中转港，开通福建居民经厦门赴金门旅游，成功申办06年奥林匹克国际合唱节和中国国际园林花卉博览会，摘取“联合国人居奖”。我们的平方根工作是：

　　（一）落实中心城市发展战略，“特、港、创、带、建”取得成效。

　　省委省政府提出构筑海峡西岸经济特区，要求厦门必须在“特、港、创、带、建”五个字上力求突破，更好地发挥龙头作用。市委作出关于“增创海峡西岸经济区建设领先优势，全面推进海湾型城市建设，加快构筑经济特区中心城市”的重要决议。一年来，我们狠抓各项工作的落实，努力在体制改革、科技创新、现代服务业、港口建设、对台工作、人居环境方面增创新优势，寻求新突破：保税区面积得以扩大，区港联动获准试点；规划建设9平方公里海空港保税联动区，并向自由贸易港区推进，加快港区泊位建设和岸线资源开发，千万标箱国际中转港建设全面启动。发展总部经济的政策效应开始显现，建行总行软件开发中心、福建中烟工业公司等一批知名企业的总部、研发销售中心向厦门聚；承接台湾光电子、石化等产业向厦门转移，对台区优势进一步凸显。完善市与区的事权、财权划分和在地统计方式，激发各区自主发展的积极性，经济竞争实力明显增强；推进事业单位人事和分配制度改革，水务、公交等公用事业实现了资本结构、运营体制的调整；放开民间资本进入外贸和公用、社会事业领域。办赛、办展的市场化运作程度提高。与周边城市开展制造业、旅游、港口、医疗卫生、信息技术服务合作，分年度投入5000万元治理九龙江排污，推动企业与漳州共建招银港区项目，城市联盟和区域协作取得进展。加强人才的培养、使用和储备，全面落实柔性引进人才政策和居住证制度，吸引海内外人员来厦创业；积极创建全国首批文明城市，明确责任，凝聚合力，全市呈现出积极进取、攻坚克难的精神风貌。

　　（二）落实宏观调控政策措施，经济增长质量明显提高

　　全面开展土地市场治理整顿，整合工业园区，提高土地集约化程度和综合利用水平，清理收回闲置土地31宗153万平方米，取消经营性房地产历史遗留项目109个300万平方米。优化投资结构，在重点项目投资中，培植税源占47%，基础设施占33%。面对全国性电力供应紧张，全市科学高度，开源节流，万元生产总值电耗减少5.6%，每度工业用电创造产值39.3元，居全国前列。金融运行质量在调控中得到提高，不良贷款比率下降4个百分点，不良资产率处于较低水平，本外币贷款余额突破1000亿元，贷款增幅高于存款增幅，贷款主要投向我市支柱产业、高新技术产业、外币型经济和重点项目，对地方经济的支持作用明显增强。

　　致力于经济增长方式的转变，大力发展高科技、高附加值、低能耗、低污染的制造业，火炬东部产业区、中科高科技产业基地动工建设，涌泉技术中心升级为国家级企业技术中心，东南融通等企业软件产业出口增长1.5倍，58家重点高新技术企业产值占全市规模以上工业产值的55.3%，电化厂等一批老工业企业完成搬迁改造，厦工工业园一期、古龙工业园一期、三圈日化等竣工投产，翔鹭纺纤、虹鹭钨丝等企业技改扩产，全市技改投资增长32.8%。落实名牌战略，新增正新、银鹭、惠尔康3个中国驰名商标和厦华彩电、厦工装载机、金龙客车、金龙旅行车4个中国名牌产品，厦华（彩电）、古龙（罐头）、伟士（体育用品）、纽威（树脂工艺品）成为国家重点培育和发展的出口名牌。支持东林电子等企业应诉反倾销案，组织企业参加重大展会，工业品产销率98.2%。产值超亿元的企业新增27家，全市工业总产值1729.2亿元，对经济增长贡献率达59%，对地方税收收入贡献率为34.9%，经济效益综合指数提高2.9%个百分点。

　　发展现代服务业，象屿保税区二期、海沧物流园区、海空港的物流中心建设取得实质性进展，“电子口岸”建设加快，通关速度进一步提高，集装箱吞吐量287.2万标箱，增长23.2%，空港国际客、货运量均居全国第四。加大对外贸企业的扶持，出口退税突破100亿元，实现“老账全退、新账不欠”，完成出口额139.5亿美元，增长32.2%，进口额突破100亿美元。扩大旅游业对周边的辐射，正式受理台胞落地办理往返大陆通行证，厦金直航旅客40.8万人次，增长1.5倍，全市游客接待量、旅游总收入分别增长21.2%和27.4%。“投洽会”等大型会展经过不断创新，影响扩大，实效增强。加强信息港平台建设，推广信息技术应用，互联网域名注册量占全国一半，服务器托管居全国第一。

　　（三）落实项目带动战略，经济发展后劲明显增强

　　加大项目招商力度，全面落实六大领域招商政策，组建全市投资信息中心，整合厦门在线招商网站，主动接洽国际相关企业，定期高度重点项目，积极解决招商项目用地问题，新获批10用地平方公里，全市合同利用外资增长57.7%，实际利用外资增长35.1%，均居全省首位，实际引进内资101亿元。目前在建和已签的内资千万元、外资千万美元以上298项目个，投资总额超过1700亿元，为“十一五”的发展积蓄了后劲。对二甲苯（PX）项目获国家批准立项，戴尔二期签订协议，汽车工业资本结构得到理顺，汽车工业城、安美光电、太古四期等项目陆续开工，石化、客车、计算机、光电子等产业集群加速形成。落实民营经济扶持政策，私营企业数量和注册资本分别增长30.3%和32.2%。

　　加大固定资产收入，突出岛外发展和对接周边，构筑中心城市基础设施框架。完善城市交通路网，环岛路全线贯通，翔安大道、海沧大道、水浏线等新建交通主干道建成通车，仙岳路中段、体育路改造等项目如期竣工。拓展对外连接通道，东通道、三泉高速厦门连接线、福厦铁路、厦漳跨海大桥等前期工作进展顺利。吸引种类资本参与海沧、嵩屿港区深水泊位，东渡港区18号泊位、10万吨级航道二期工程竣工投入使用。

　　（四）落实统筹协调要求，城乡经济社会全面进步

　　创新“三农”工作机制，通过扶持龙头企业、发展订单农业，推动规模经营，带动农业结构的优化，建成20个无公害蔬菜、畜禽、水产基地和1.5万亩现代农业示范基地。新增3家国家级农业产业化龙头企业，全市龙头企业销售收入、出口和税利分别增长34%、41%和23%，带动8.5万户农民增产增收，订单农业达12万亩，占耕地面积的33.1%。市财政1亿多元，加快农村义务教育和公共卫生建设，已开工项目87个，其中27个竣工并投入使用。分年度投入2.5亿元，启动自来水进村入户工程，已有4万农民受益。建成300座水冲式公厕，完成通村通道路硬化83公里，开通8条农村客运线路。建成150口抗旱大井，适时开展人工增雨，治理后溪流域，加固47座水库和堤防等水利设施，改善灌溉面积3.4万亩。加强农村富余劳动力培训，引导农民自主创业，实现转移3.3万人。加快农村城市化，禾山、杏林完成“镇改街”。深化农村税费改革，减轻农民负担4000多万元。金融机构对农业贷款增加7.7亿元。继续实行市级领导带队挂钩帮扶，31个经济薄弱村生产生活条件明显改善。

　　加大对社会事业投入，全社会投资增长43%，其中对教育和卫生投资分别增长84%和26.5%，文化艺术中心、嘉庚体育馆、市疾病控制中心等一批项目进展顺利，理工学院、华侨大学集美校区建设和一中扩建、双十中学新校区工程如期开工，国家会计学院、城市大学一期、外国语学校海沧分校等工程如期竣工，新建成7所社区配套学校，全面普及高中阶段教育，基础教育课程改革走在全国前列。成功举办第二届厦门（夏新）国际马拉松赛、第六届全国舞蹈比赛、海峡两岸歌仔艺术节活动。编纂出版建国以来首部《厦门市志》，公布第五批市级文物保护单位，广播电视、新闻出版、报业改革取得新进展。整合医疗卫生资源，推动中山医院兼并铁路医院、第二医院兼并集美医院，妇幼保健院顺利搬迁，初步建成医疗紧急救援立体网，启动重大疾病防治技术攻关计划，公共卫生体系更加完善。严格人口与计划生育管理，出生人口政策符合率达到97%。注重国防动员和后备力量，人防设施得到加强。外事侨务、民族宗教、地震气象、知识产权保护等工作，都取得新的成绩。

　　推进生态城市建设，整治铁路沿线、工地扬尘、油烟扰民和燃煤污染，城市空气质量、水质、噪音和污染物排放各项控制指标，均优于国家环保城标准。污水管网建设加快，污水集中处理率提高到78%。继续美化城市景观，造林更新6000亩，林相改造1.1万亩，新增绿地350公顷，忠仑公园、同安东溪公园等建成开放，整治裸露山体，关闭一批采矿点，完成828公顷水土流失治理。新建住宅260万平方米，城市居民人均居住面积提高到21.8平方米，莲坂、刘厝、祥店等旧城旧村改造进展顺利，“烂尾楼”工程基本消化。

　　（五）落实执政为民宗旨，群众生活进一步改善

　　广开就业门路，落实灵活就业鼓励措施，建设区级中心劳动力市场，在全国率先建立了覆盖市、区、街（镇）、居（村）四级就业信息网络。通过“1＋1群”创业培训带动就业，对“4050”人员、困难职工实施就业与再就业援助，对大中专毕业生职业见习和技能培训实行财政补贴，全年新增就业20万人。成立劳动争议仲裁院，劳动关系三方协商机制延伸到区，打击非法职介等违法行为，积极维护外来员工合法权益，清理拖欠工程款2.3亿元、农民工工资2600多万元，促进劳动关系和谐稳定。

　　健全社会保障体系，市本级全年社会保障投入达6.6亿元，实施被征地人员、转产渔民和事业单位职工基本养老保险办法，社保覆盖面扩大。推行新型农村合作医疗制度，农民参保率　由51.1%提高到80.6%。城乡低保基本实现“应保尽保”。企业退休人员社会化管理率达到96.1%。出台政策帮助贫困家庭解决就医、子女就学，支持开办“爱心超市”、“博爱超市”，重视残疾人和老龄事业，成为全国社区红十字服务示范市、无障碍设施建设示范城。

　　更加关注民生，基本完成10项为民办实事项目。重视粮食安全，增加地方粮油储备。全面实施食品卫生监督量化分级管理，加强农产品检测检疫，控制了餐桌污染，防止了禽流感疫情的发生。社区服务设施进一步改善，新型社区组织初步形成。健全市民基础服务信息系统，启用社会保障卡和劳动保障咨询电话，完善小额支付E通卡。纠正医药购销和医疗服务中的不正之风，严格药品认证，确保市民用药安全，新增社区医保定点医疗机构20家，开通医保定点零售药店23家。规划建设10万平方米外口公寓，启用进城务工人员活动中心。积极扶持民办教育，有效解决外来员工子女就学。改革两桥收费办法，经济适用房建设向岛外延伸。

　　积极创建“平方厦门“，落实社会治安综合治理责任制，实行110.119.122三台全合一，建成171个社区警务室，按照预案建立了统一高效的应急处置体系。开展缉枪、治爆、禁毒专项行为，刑事犯罪高发势头得到遏制，破案率提高13.5%个百分点，人民群众对社会治安满意率达96.3%，居全省第一。加强安全生产监管，严格整治事故隐患，事故发生数和经济损失额均下降50%以上。继续实施“四五”普法，加强基层人民调解组织建设，妥善处理了一批人民内部矛盾纠纷和群体性事件。

　　（六）落实政府自身建设各项措施，行政能力得到提高

　　严格依法行政，贯彻执行《行政许可法》，组织开展行政许可规定、实施主体、审批事项和行政收费的清理工作，废止和修订部分市政规章和规范性文件。成立城市管理行政执法局，整合海洋、渔业行政执法力量，城市管理行政处罚权实现相对集中。提高预算管理、公共管理能力，取消非财政性资金项目的立项审批，14个单位66项项目试行网上审批，推行前置审批告知承诺制，建设项目审批时限压缩到45个工作日。全面落实经营性土地使用权“招拍挂”出让制度，完善经评审的最低价中标和业主招标，规范了市场秩序，堵塞了管理漏洞，财政投资的工程项目造价降低23.1%。全面清理行政事业性收费，教育、卫生收费通过国家专项验收。加强审计和监察工作，整肅财经纪律，查出违规金额8600万元，查处违法违纪案件197件199人。

　　尊重群众的知情权、监督权，扩大政务公开范围，就征地拆迁补偿安置、鼓浪屿旅游票制改革、教育收费调整等问题召开市民听证会，坚持公民旁听市政府常务会议制度。开通968123市长专线电话，市政府及部门领导直接受理市民反映的问题，抽样满意率97.6%，市民来信、个访分别下降22.7%和23%。认真办理人民代表议案、建议和政协委员提案、建议，满意率分别达93.7%和95.6%。

　　各位代表！过去一年取得的成绩，是全面贯彻落实中共中央、国务院和福建省委、省政府各项方针政策的结果，是中共厦门市委正确领导的结果，是全市广大干部群众齐心协力、奋发图强的结果，得益于历届市委、市政府打下的良好基础，离不开市人大、市政协的支持和监督。在此，我谨代表市人民政府，向全市人民，向各民主党派、工商联、人民团体和社会各界人士，向离退休老同志，向中央和省驻厦单位，向驻厦部队、武警和公安民警，向所有来厦的投资者和劳动者，向关心支持厦门的港澳同胞、台湾同胞、海外侨胞和国际友人，表示崇高的敬意和衷心的感谢！

　　当前，我市经济社会和政府工作仍存在不少困难和问题，主要是：土地供需矛盾突出，土地调整、征地拆迁难度大，40%的重点项目受到影响；用电紧张局面依然比较严峻，原材料、农贸等价格涨幅较大，给部分企业和群众的生产生活带来一定影响；现代服务业相对滞后，影响中心城市功能的发挥和核心竞争力的形成；城市基础设施、社会事业发展进入新一轮大投入时期，政府债务处于偿还高峰，需要安排的出口退税资金大幅增加，财政面临前所未有的压力；农民增收难度加大，部分群众生活困难，社会保障任务很重；企事业单位改制和征地拆迁过程中各种矛盾仍然存在，群体性上访等社会稳定问题不容忽视；城市管理跟不上城市发展，市容管理有所松懈，违章违法建设仍然存在；山林火灾时有发生，交通事故伤亡较多；政府职能转变还不能适应市场经济发展的要求，一些部门行政效率不高，服务意识不强，个别政府工作人员缺乏责任心，存在形式主义、回避矛盾、推诿扯皮现象。对这些问题，我们一定要高度重视，千方百计克服困难，努力加以解决。

　　二、05年的任务

　　05年，我们既面临较好的国际国内发展环境，又面临着许多新的困难和竞争压力，既要全面完成“十五”计划预期目标，又要为“十一五”的发展奠定基础，任务更为艰巨、更加繁重。政府工作的总体要求是：以邓小平理论和“三个代表”重要思想为指导，深入贯彻党的十六大和十六届三中、四中全会精神，认真贯彻省委、市委一系列决议决定，坚持以科学发展观统领全局，全面落实建设海峡西岸经济区的决策部署，致力提升城市核心竞争力，着力推进改革开放，加快调整经济结构，转变经济增长方式，提高经济效益，增强发展后劲，改善人民生活，构建和谐社会，增创海峡西岸经济区建设领先优势，全面推进海湾型城市建设，加快构筑经济特区中心城市，实现经济社会更快更好发展。主要预期目标建议为：生产总值增长15%，地方级财政收入同口径增长17%，城镇居民人均可支配收入和农民人均纯收入分别增长10%和8%，城镇登记失业率控制在4.3%以内，居民消费价格指数103左右，人口出生率控制在10.5‰以内。在实际工作中要加倍努力，力争完成得更好。

　　按照上述要求和目标，今年要扎实做好五个方面工作：

　　（一）项目带动，优化结构，不断提高产业竞争力

　　把经济增长的重点坚定地放到提高效益上来，主要依靠科技进步和产业配套，实现制造业素质的提升和规模的扩张。力促尔戴电脑二期、对二甲苯（PX）等大型项目尽早动工，加快火炬东部产业区、汽车工业城和国家半导体照明工程产业化基地建设，确保腾龙特种树脂、金龙联合新厂等项目竣工达产，抓紧完善工业园区基础设施，引导中小企业进入专业园区，促进产业链延伸和产业集聚。着力发展产业科技，支持企业自主知识产权的研发创新，大力引进国内外先进技术，筛选确定重点扶持的科技项目，组织好银企、银技对接，促进科技成果转化为实际生产力。利用高新技术提升传统产业，继续推动中药厂等岛内工业企业搬迁改造。加强工业经济运行的分析调控，做好煤电油运和重要原材料的调度，及时帮助解决生产经营中遇到的困难和问题。继续实施名牌战略，支持生产出口型企业开发国内市场，培育和做大“厦门造”名品牌。

　　立足于提高城市综合竞争力，主动对接服务周边，做大现代服务业。发展港口物流业，确保区港联运通过国家验收并启动试点，建设海空保税联运区，建成海沧物流园区，吸引航商增辟航线加密航班，降低口岸营运成本，拓展江西、粤东、湘南等纵深腹地，吸引更多的货物在厦门集散，推进国际中转港建设，把厦门构建成为海峡西岸航运物流中心。发展旅游会展业，抓紧建设喜来登等十大高星级酒店和日月谷温泉度假村扩建等十大休闲娱乐景点，组织好福建居民赴金门旅游，与泉州、漳州、龙岩和武夷山互为客源市场，协调建设“无障碍”旅游区，稳步实施鼓浪屿旅游票改。发展商贸批发业，加快SM、明发商业广场等重点商业区建设，支持海沧、翔安、同安区新建大型专业批发市场，形成为本市和周边产业发展配套服务的商品交易中心。发展文教卫体产业，拓展中介服务业，鼓励社会力量办会办展，促进旅游、会展、商贸、文体的互动，扩大消费总量。发展金融证券保险业，努力构建区域性金融中心，推进城市商业银行、农村信用社改革，更多地吸引国际和台湾金融企业来厦设立机构，推动港务集团等企业境内外上市融资，支持期货业务发展，建设金融征信体系，巩固厦门金融安全区。

　　坚持工业反哺农业、城市支持农村，以建设高效特色、绿色生态农业为目标，推进农业产业化、集约化经营。在政策、融资、研发和争创名牌等方面扶持农产品加工龙头企业，力争产值突破100亿元。完善土地流转机制，鼓励企业租用农民土地、聘用当地农民，进行规模开发，扩大订单农业面积，增强加工出口带动种养的能力。扩大种苗繁育基地和现代农业示范基地，发展休闲农业、休闲渔业，提高农业综合效益。

　　（二）突破瓶颈，破解难题，全面推进中心城市建设

　　征地拆迁是影响当前重点工程建设的一大难题，躲不开、绕不过、等不得，要依据法律法规，维护各方权益，研究具体有效的办法切实加以解决。抓紧完成土地利用总体规划修编，积极用地指标，盘活存量土地，落实土地与项目对接，确保基础设施和税源项目的用地需要，集约使用土地，合理调整建筑容积率，提高单位面积土地的投资强度和使用效益。总结经验教训，优化用电预案，优先保障居民生活和重点企业、重点部位用电，倡导节约用电，鼓励企业用好自备电源，做好计划外电量购买工作，推动海沧500千伏、翔安220千伏等输变电项目建设。续建坂头水库扩容工程，汀溪水库和湖国这水库储备水源，着手开辟第二水源。发展新兴节能产业，推进循环经济立法，建设试点园区，进一步降低万元生产总值综合能耗，使有限的资源发挥最大效益，力争我市循环经济走在全国前列。

　　加快构筑中心城市基础设施框架，开工建设东通道，推动翔安新区开发建设，展开海湾型城市的东翼。建设演武路至机场快速路、杏林至高崎公铁大桥，架构城市南北交通大动脉。改造疏港路，续建仙岳路、县黄路、集美大道、海湾大道等城市主干道，完成环湖里大道、吕岭路等与环岛路的网状连接，做好福厦铁路的开工和厦漳跨海大桥、龙厦铁路、深厦铁路、机场三期的前期工作，推进东渡、海沧、嵩屿港区和航道扩建工程。加快信息港建设，开发基础数据库，推动信息技术在各行业的普及应用，建设服务全省的公共信息平台，，把厦门建设成为海峡西岸信息网络通讯枢纽、信息资源集散地和信息技术服务中心。

　　推进区域经济协作向纵深发展，在基础设施、产业分工、社会事业以及联合招商方面与周边城市广泛协作。动工建设三泉高速厦门连接线，同漳州市一道加快厦门港的组合与开发建设，在水资源开发、污水处理、交通干道和散装泊位建设方面，推动厦、漳城市联盟取得实质性进展。拓展与龙岩、三明、南平的山海协作，推进闽粤赣十三市经济协作，发挥内联企业、各地驻厦机构的作用，密切与珠三角、长三角和内陆省份的经济联系。继续做好与西藏、宁夏、重庆、新疆等地对口支援和帮扶工作。

　　（三）深化改革，扩大开放，增强发展的动力和活力

　　牢记经济特区改革创新、先行先试的使命，坚持不懈地解放思想、锐意进取，用改革的思维、法治的原则、市场经济的手段，破除体制性障碍。进一步下放事权、财权、审批权，让特别区放开手脚，择优发展。成立国有资产管理委员会，整合国有资产投资运营机构，扩大授权经营。加快市属国有企业改革和集体休业改制，主动争取与同行业国际领先企业实现战略合作。深化投融资体制改革，进一步放开基础设施、市政公用、社会事业特许经营权，再推出一批项目交由市场主体运营。组建社会事业发展投资公司，实行“管办分离”，推进事业单位配套改革，逐步推广年薪制。实施人才强市战略。用政策吸引更多的高等院校、科研院所、研发机构来厦创业，促进院校培养与企业需求对接，培养引进种类高层次和紧缺人才，壮大高素质企业家和高技能员工队伍。放开领域。引优扶强，大力发展民营经济，重点扶持科技型、贸易型和服务型民营企业，鼓励参与国有和集体企业的改制重组，完善政策性担保机构，定期组织“银企对接”，拓宽融资渠道，发挥工商联和行业协会的作用，为民营经济加快发展创造良好环境。

　　坚持出口带动战略不动摇，积极开拓国际市场，保持外向型经济的领先优势。及时足额兑现地方承担的出口退税，促进对外贸易较快增长。整合口岸物流信息平台，实现地方与国家“电子口岸”的对接，推动加工贸易联网监管，规范外贸经营和报关代理市场秩序，扩大信用通关，提高口岸便利化程度。加强对我国加入世贸组织“后过渡期”政策的研究，做好产业损害预警，协助企业应对技术壁垒和反倾销应诉。引导企业优化出口商品结构，扩大高新技术产品、机电产品和软件产品出口，促进加工贸易的转型升级。抓住东盟自由贸易区启动的契机，拓展与周边国家的经贸合作。出台鼓励政策，积极做大进口，重点培育电子、工业原辅料、日用消费品等商品的进口分拨中心。支持企业“走出去”，到境外设立贸易网点和投资生产性企业，扩大境外工程承包和劳务输出。

　　加大招商力度，为“十一五”发展积蓄后劲。把握服务贸易领域市场准入放宽和门槛降低的有利时机，深化内地与港澳建立更紧密经贸关系安排（CEPA）框架下厦门与港澳的经贸合作，注重引进服务贸易项目。跟踪国内外企业集团、上市公司的投资意向，做细做深项目策划，采用联合并购、股权招商和以存量引增量的“零地招商”等多种方式，大力引进光电子、生物医药、软件等行业龙头企业。深化鹭江道、金尚路、湖滨北路三大总部经济带规划，完善鼓励政策，吸引国内外企业来厦设立区域总部、研发机构和采购中心。不断改革“投洽会”办会机制，发挥亚欧经贸对话平台的作用，加快向国际投资博览会迈进。办好第六届世界同安联谊会，做好华侨、华人、侨眷的工作，加深与国际友城的合作，密切与境外掆机构的联系，建立国内外招商互动网络，维护投资者合法权益，吸引更多跨国公司和知名企业在厦投资兴业。

　　拓展厦台交流合作，凸显对台区位优势。积极争取扩大台商投资区，主动加强与台湾工商团体的沟通联系，继续办好“台交会”，促进新一轮产业对接。发挥台商协会的作用，及时解决台商在生产生活中遇到的困难和问题。扩大厦金直航成果，使厦门成为台胞往返祖国大陆的主要口岸。拓展厦金货物直接往来通道，办好大嶝对台小额贸易市场。利用“厦－金－台”海空联运优势，做大台湾旅游市场，办好第八届海峡两岸旅游业联谊会。发挥人文地理优势，促进厦台文化、教育、体育、卫生等方面的交流与合作。

　　（四）统筹协调，加大投入，促进城乡经济社会全面发展

　　立足本市，服务周边，努力构建闽西南文化教育、医疗卫生中心。对已经安排的社会事业项目要逐项精心落实，确保高质量完成。加快建设集美文教区和翔安文教区，迁华厦学院和高级技工学校，鼓励创办工科院校、民办高校，完成优质高中和中等职业中心校的扩建。整合教育资源，强化薄弱校建设，促进基础教育均衡发展。巩固义务成果，全面实施素质教育，启动高中课程和新一轮基础教育课程改革。加快建设中医院新院和市疾病控制中心，扩建第一医院、中山医院和第三医院，开展精神心理疾病康复指导，完善公共卫生和立体医疗急救体系。提倡婚前体检。促进优生优育，强化流动人口计生管理服务，治理性别比偏高总量　。推进文化艺术中心和嘉庚纪念馆建设，开工建设小白鹭艺术中心剧场，扶持爱乐乐团，培育闽南地方戏剧，鼓励文艺精品创作，举办纪念抗日战争塈世界反法西斯胜利60周年合唱节，积极筹办06年奥林匹克国际合唱节，开播并办好海峡卫视和闽南之声广播，发展广播电视、报纸等传媒产业。继续抓好嘉庚体育馆、国际网球中心和工人体育馆等项目建设，办好第17届市运会、国际铁人三项赛。主动联合周边城市策划宣传，扩大厦门国际马拉松赛品牌的影响，推动全民健身运动广泛开展。

　　坚持以增加农民收入为目标，继续改善农村生产条件和生活条件，深入落实扶持龙头企业、扩大订单农业、加快富余劳动力转移三大举措，扎实推进城市化进程中农民的“居住社区化、从业非农化、资产股份化、福利社保化”。做好“村改居”集体经济组织改制，鼓励农民通过参股建设批发市场、外口公寓、通用厂房等增加收入。引导未升学的初高中毕业生直接进入职业学校，继续办好本地农民工专场招聘会，确保转移富余劳动力1万人以上。加大财政转移支付，巩固农村税费改革成果。市财政继续投入1亿元，推进未完成的60个农村义务教育和公共卫生项目建设，扩大有线电视覆盖面，提高自来水入户率，续建一批水冲式公厕和垃圾处理场，基本完成通过道路硬化。关心支持老区基点村建设。继续抓好重点流域治理，加固海堤，修复病险水库，续建高崎闽台渔轮避风港，提高减灾能力。加强动植物疫病检测与防治，防控高致病性禽流感等疫情发生。

　　进一步改善人居环境，完善商品房供应市场体系和中低收入市民房保障体系，促进供求结构基本合理，努力保持房价基本平稳，加大土地供应量，抓好经济适用房、廉租房和外口公寓的建设。启动新店、杏北新城建设，推进厦港、百家村旧城改造，加大对违法建筑的查处力度。巩固西海域整治成果，保护东海域生态环境，完成员当湖清淤整治，做好无居民岛屿的保护和利用。继续整治裸露，治理水土流失，加强莲花国家森林公园的保护，新建东坪山市花公园，新增城市绿地350公顷，继续推进林相改造，扩大城市生态林、风景林。防治扬尘污染，治理工业废气和机动车尾气，力争用三年时间把空气质量恢复到年平均“优”的水平。加快建设垃圾分拣中心和东部固体废弃物处理中心，提高垃圾处理率。扩建市污水处理二厂，做好中水综合利用。集中力量筹办第六届中国国际园林花卉博览会，把“园博会”建设成为主题突出、特色鲜明的旅游观光新景点、休闲娱乐新公园。

　　（五）争创文明城市，落实以人为本，努力构建和谐社会

　　以建设廉洁高效的政务环境、公正公平的法治环境、规范守信的市场环境、健康向上的人文环境、安居乐业的生活环境和可持续发展的生态环境为目标，创建首批全国文明城市。严格对照《文明城市测评体系》，全市动员、全民动手，分线落实、分片检查，社会各界献计出力，群众参与点评监督，全面整治市容环境、交通安全等薄弱环节，重点解放群众关注的突出问题。加强社区建设，开展公民道德教育，增强诚信守法意识，以医疗卫生、帮扶救助、就业服务、退休人员服务、老年服务为重点，完善社区服务功能，建设文明安全和谐社区。深化军民共建、警民共建内涵，增进军政军民团结。通过创建活动，努力提高市民的文明素质和生活质量，提高城市的综合竞争力。

　　扩大创建“平安厦门”成果，不断提高人民群众对社会治安的满意率。严厉打击涉黑、涉枪、涉爆等突出刑事犯罪，保护人民群众生命财产安全。严厉打击“六合彩”等赌博活动，铲除社会公害。严厉打击制毒贩毒犯罪行为，绝不让厦门成为贩毒通道和毒品危害的灾区。坚持“打防结合、预防为主、专群结合、依靠群众”，完善社会治安综合治理工作机制和治安防控体系，继续加强校园周边治安整治，及时解决群众反映的治安热点问题。严密防范和严厉打击境内外敌对势力的破坏、渗透活动。进一步整顿和规范市场经济秩序，打击走私贩私、制假售假、偷逃骗税、商业欺诈等经济违法活动。调度重视和正确处理人民内部矛盾，发挥调解、仲裁等机构的作用，构建矛盾纠纷多元化解决机制，认真贯彻国家《信访条例》，继续办好市长专线，直接聆听市民意见，畅通社情民意反映渠道，坚持领导干部约访、下访制度，及时妥善处理群众来信来访反映的问题。落实行政首长事故责任追究制，完善重大自然灾害、事故灾难、公共卫生、社会安全等突发事件处置预案，增加预警预报投入，提高应急处置能力，加强对事故隐患的排查整改和专项整治，坚决遏制重特大安全事故的发生。

　　始终把就业作为民生之本，通过产业发展、扶持部分劳动密集型企业，不断扩大就业规模，争取全年新增就业15万人。发挥创业促就业的作用，通过小额贷款担保、创业培训扶持自主创业。完善就业服务体系，鼓励灵活就业，继续对“4050”等人员实施就业援助，保证失业人员再就业率达到60%以上。做好高校毕业生就业指导和信息服务，妥善安置退伍和转业军人。

　　进一步完善社会保障体系，减少人民群众的后顾之忧。强化社保基金的征缴和监管，逐步拓展筹资渠道，做实基本养老保险个人账户，确保基金安全。重点解决被征地人员、事业单位职工基本养老保险，提高灵活就业人员和外来员工的社会保险参保率。实施新的工伤保险条例，确保工伤患者得到及时救治。完善基本医疗保险，从今年起政府对参保的困难人员实行医疗费补助制度。政府为全市农民投保，推行大病医疗保险，实行农村计生奖励扶助。鼓励社会力量参与兴办福利事业和慈善事业，切实保障妇女、儿童、老年人和残疾人的合法权益。深入开展扶贫帮困，动员和依靠社会力量与政府一道，对群众的特殊困难给予救助，保证每个贫困家庭的子女才有接受义务教育和职业教育的机会，保证每个考上大学的学生都不会因贫失学。

　　注意倾听群众的意愿和呼声，诚心诚意地办实事、做好事，解决关系人民群众切身得益的实际问题。组织力量，加大投入，重点解决好食品安全、药品安全和交通安全问题。大力推广放心食品工程，建设食品安全信用体系，治理餐桌污染。强化药品监管，保证用药安全。严格执行道路交通法规，提高市民交通安全意识，完善交通警示设施，尤其要保障中小学生的交通安全，努力减少交通伤亡事故。优化公交线路，改建枢纽站点，新设65座公交候车亭，方便群众出行。开通镇、村信息服务网络，取消进城农民工子女义务教育借读费，成立外来员工劳动就业和培训、娱乐、婚庆服务中心，不断改善外来员工的生活条件。

　　三、全面提高行政能力，建设对人民调度负责的政府

　　市委九届九次全会提出了贯彻落实《中共中央关于加强党的执政能力建设的决定》的若干意见。在新的一年，我们要坚持不懈地建设对人民调度负责的政府，始终坚持发展为大、发展为重、发展为先，始终坚持人民为大、人民为重、人民为先，围绕行政能力的全面提高，把政府自身建设摆到更加突出的位置，切实抓紧抓实、抓出成效。

　　提高科学决策能力。认真贯彻落实市委的各项决议决定，在具体的工作决策过程中充分发挥民主，善于集中民智，建立健全并认真实施重大事项的专家咨询、社会公示、听证和集体决策制度，认真听取人民代表、政协委员和各民主党派的意见，推进决策民主化和科学化。本着对厦门的发展负责、对全市人民群众负责，着眼长远，着力根本，勇于积极探索，敢于先行先试，把各项事业不断推向进步。轻编制“十一五”规划为契机，加强对关系厦门长远发展和国计民生重大问题的调研，正确认识和把握厦门的发展阶段和发展水平，正确认识和把握厦门的发展定位和发展战略，正确认识和把握厦门的发展模式和发展重点，正确认识和把握厦门的发展优势和制约瓶颈，制订一部能切实指导厦门今后更快更好发展的规划，争取更多的项目纳入国家发展规划。

　　提高依法行政能力。认真落实《行政许可法》、《全面推进依法行政实施纲要》确定的各项任务，大力增强政府工作人咒法制意识，严格按照法定权限和法定程序行使权力、履行职责，依法处理经济社会事务。发挥经济特区和较大城市立法权优势，营造更好的福分秩序和发展环境。为发展和维护公众的整体利益和长远利益，公正执法、文明执法、大胆执法，敢于碰硬、敢破难题，依法排除强买强卖、无理阻工等违法现象，善于运用法律和强有力的思想工作手段，解决经济社会发展中各方面的利益矛盾。落实行政执法责任制，加强行政执法监督，继续做好行政复议和应诉工作，自觉接受人大及其常委会的工作监督和法律监督、人民政协的民主监督以及新闻舆论、社会公众的监督，实行行政过错责任追究，规范约束行政权力，切实做到执法有依据、有权必有责、用权受监督、违法受追究、侵权须赔偿。

　　提高公共服务能力。加强行政机关交通建设，实行政府及其部门的绩效评估以及机关工伤人员的绩效考评，把考评结果作为奖惩晋级的重要依据，在用人标准上发挥导向作用。深化行政审批制度改革，全面实行告知承诺制度，减少事前审批，加强事后监管，建设网上审批服务系统，扩大范围、简化环节，提高审批效率和服务水平，切实转变政府职能。各级政府工作人员要增强忧患意识和责任意识，珍惜当前的大好发展时机，敢抓敢管、敢于负责，不推诿、不扯皮，狠抓各项任务的落实。要进一步加强业务学习，积极追踪和借鉴先进城市发展的成功经验，善于捕捉国际信息，创新工作思路，不断提高政府发展经济和管理社会事务的能力。

　　提高拒腐防变能力。贯彻落实《中共中央关于建立健全教育、制度、监督并重的惩治和预防腐败体系实施纲要》，严格执行领导干部“四项纪律、八项要求”，严格遵守不准收受礼金和有价证券、不准“跑官要官”、不准利用职权谋取私利、不准参与赌博、不准借婚丧嫁娶之机敛财等五项规定。落实反腐倡廉“六个机制”，强化“一岗双责”，认真执行任期经济责任审计和述廉制度。各级领导干部要以身作则，不仅管好自己，还要管好配偶、子女和身边的工作人员，决不做有损党和政府形象的事，决不做有损人民利益的事。加强部门和行风建设，完善土地的拍挂、工程招投标、政府采购、产权交易四项制度，坚决杜绝政府工作人员以任何方式插手资源、项目、投资审批和工程承包，决不允许任何人利用职权谋取私利，保证权力干净运行。对违法乱纪者，从严惩处，决不手软，决不姑息。进一步加强部门预算管理，扩大绩效审计范围，强化审计监督。弘扬勤俭办事、艰苦奋斗的作风，严格控制出国考察、大型会议、公务用车等财政支出，削减一般性开支，继续压缩行政经费5%。全体政府工作人员要常修为政之德、常思贪欲之害、常怀律己之心，勤于自省、善于提醒、敢于批评，争当勤政高效、清正廉洁的表率。

　　全面实现今年政府工作的各项目标，重在责任，贵在落实，我们要进一步增强责任意识，切实履行岗位职责，不断提高行政能力和水平，担负起人民赋予我们的重任。维护群众的利益高于一切，关心群众的疾苦重于一切，解决群众的困难先于一切。凡是得民心、顺民意的事情，都要竭尽全力地去做；凡是失民心、背民意的事情，都要雷厉风行地去改；凡是对人民群众作出的承诺、安排，都要说到做到、说好做好，确保及时兑现。要弘扬求真务实精神，大兴求真务实之风，坚持办实事、重实干、求实效，不急功近利，不搞形式主义，不做表面文章。大兴调查研究之风，压缩会议、减少文件，提倡讲真话、讲实话，开短会、开有用的会，挤出时间到基层去，到群众中去，到矛盾多困难大的地方去，解决实际问题。政府全体工作人员要切实对厦门发展调度负责、对220万厦门人民高度负责，倍加珍惜聚精会神搞建设、一心一意谋发展的良好氛围，时刻保持奋发有为的蓬勃朝气、开拓创新的昂扬锐气、清正廉洁的浩然正气，通过一桩桩、一件件扎扎实实的工作，充满感情、千方百计、全力以赴把广大人民群众的利益维护好、实现好、发展好，使政府的各项工作经得起实践的检验、群众的检验和历史的检验。

　　各位代表！让我们更加紧密地团结在以胡锦涛同志为总书记的党中央周围，高举邓小平理论伟大旗帜，全面贯彻“三个代表”的重要思想，在中共厦门市委的领导下，求真务实，埋头苦干，确保完成经济社会发展各项任务，为全面完成“十五”计划，为创造厦门更加美好的明天而努力奋斗！
